# Improvement in Image Quality of Low-Dose CT of Canines with Generative Adversarial Network of Anti-Aliasing Generator and Multi-Scale Discriminator

**DOI:** 10.3390/bioengineering11090944

**Published:** 2024-09-20

**Authors:** Yuseong Son, Sihyeon Jeong, Youngtaek Hong, Jina Lee, Byunghwan Jeon, Hyunji Choi, Jaehwan Kim, Hackjoon Shim

**Affiliations:** 1 Department of Computer Engineering, Hankuk University of Foreign Studies, Seoul 02450, Republic of Korea; yuseoung.son@gmail.com (Y.S.); bhjeon@hufs.ac.kr (B.J.); 2 Brain Korea 21 Project, Graduate School of Medical Science, Yonsei University College of Medicine, Seoul 03722, Republic of Korea; sihyeonjeong552@gmail.com (S.J.); qqwwdj@yonsei.ac.kr (J.L.); 3 CONNECT-AI Research Center, Yonsei University College of Medicine, Seoul 03760, Republic of Korea; hjshim@yuhs.ac; 4 Department of Veterinary Radiology, College of Veterinary Medicine, Konkuk University, Seoul 05029, Republic of Korea; chj1824@naver.com (H.C.); jaehwan@konkuk.ac.kr (J.K.); 5 Canon Medical Systems Korea Medical Imaging AI Research Center, Seoul 06173, Republic of Korea

**Keywords:** denoising, low-dose CT(LDCT), cycle-GAN, deep learning, veterinary imaging

## Abstract

Computed tomography (CT) imaging is vital for diagnosing and monitoring diseases in both humans and animals, yet radiation exposure remains a significant concern, especially in animal imaging. Low-dose CT (LDCT) minimizes radiation exposure but often compromises image quality due to a reduced signal-to-noise ratio (SNR). Recent advancements in deep learning, particularly with CycleGAN, offer promising solutions for denoising LDCT images, though challenges in preserving anatomical detail and image sharpness persist. This study introduces a novel framework tailored for animal LDCT imaging, integrating deep learning techniques within the CycleGAN architecture. Key components include BlurPool for mitigating high-resolution image distortion, PixelShuffle for enhancing expressiveness, hierarchical feature synthesis (HFS) networks for feature retention, and spatial channel squeeze excitation (scSE) blocks for contrast reproduction. Additionally, a multi-scale discriminator enhances detail assessment, supporting effective adversarial learning. Rigorous experimentation on veterinary CT images demonstrates our framework’s superiority over traditional denoising methods, achieving significant improvements in noise reduction, contrast enhancement, and anatomical structure preservation. Extensive evaluations show that our method achieves a precision of 0.93 and a recall of 0.94. This validates our approach’s efficacy, highlighting its potential to enhance diagnostic accuracy in veterinary imaging. We confirm the scSE method’s critical role in optimizing performance, and robustness to input variations underscores its practical utility.

## 1. Introduction

Computed tomography (CT) is a non-invasive, cross-sectional view of the body carried out by transmitting X-rays through the subject, which can provide information on the size, shape, and location of lesions, as well as tracking disease metastasis. However, compared to medical imaging techniques such as MRI and ultrasound, there are ongoing concerns about radiation exposure to subjects between imaging [1]. Compared to humans, animals may be thought to require lower doses due to their smaller size, but higher doses are required to see their small anatomy at high resolution. In addition, animals are less able to communicate than humans, which can make radiation exposure more dangerous. Compared to standard-dose CT (SDCT), low-dose CT (LDCT) offers the advantage of reducing medical exposure by minimizing radiation dosage. However, this reduction comes at the cost of a lower signal-to-noise ratio (SNR), leading to diminished image quality. Consequently, there is a pressing need for alternative methods capable of producing high-quality images under low-dose conditions in animal CT imaging. Recently, super-resolution and denoising techniques using deep neural network learning have been proposed to overcome this quality degradation problem [2,3,4].

One task that has benefited greatly from advances in deep learning technology is denoising. Previous deep learning-based image quality improvement research has used per-pixel loss functions based on mean square error (MSE) [5]. While MSE has maintained high scores on measures such as peak signal-to-noise ratio (PSNR) by focusing on the similarity between pixels, it has limitations such as lack of high-frequency detail and poor quality in perceptual details [6]. In addition, convolutional neural network (CNN)-based models require pairs of input data for training. Deep denoising networks are not a reliable solution for unpaired noisy images, but it is difficult to obtain such data for medical images such as CT scans. To address this limitation, many unsupervised deep learning models have been proposed.

Unsupervised learning, where the model identifies patterns or relationships in data without labeled training data, is particularly useful for tasks with scarce data. Cycle-consistent generative adversarial networks (CycleGAN) [5] are a typical model in this category, using two GANs and cycle consistency loss. Various modifications to the generators, discriminators, and loss functions have been proposed to improve CycleGAN’s performance, including the application of AdaIN [2] from StyleGAN and the addition of style reconstruction and perceptual losses [5,7].

Our method introduces several key innovations to address the limitations of traditional CT imaging. First, we enhance the generator network architecture by incorporating BlurPool [8] layers to mitigate aliasing effects during down-sampling, thus preserving high-frequency details essential for accurate medical imaging. Aliasing occurs when high-frequency components of an image are misrepresented during down-sampling, leading to artifacts and loss of detail. This issue is particularly problematic in medical imaging, where precise details are crucial for accurate diagnosis [9]. Second, we integrate the hierarchical feature synthesis (HFS) module [10] into our residual network. HFS effectively aggregates residual features, preserving high-frequency details and improving feature representation. This module, combined with spatial and channel squeeze and excitation (scSE) blocks, enables the model to focus on relevant spatial information and enhance segmentation performance. 

Additionally, our approach employs a multi-scale discriminator network that evaluates images at various resolutions. This multi-scale strategy improves the discriminator’s ability to capture fine details and patterns, resulting in superior image reconstructions compared to single-scale approaches.

Our contributions are as follows.

1. We propose an enhanced generator network architecture incorporating BlurPool layers to mitigate aliasing effects and improve image quality under low-dose conditions.
2. We apply the hierarchical feature synthesis (HFS) module to leverage spatial information effectively without loss, enhancing feature extraction and representation in medical imaging.
3. We utilize a multi-scale discriminator to improve the discrimination of fine details, such as edges and patterns, resulting in higher-quality image reconstructions.

## 2. Related Work

### 2.1. GAN-Based Solutions for CT Denoising

In the field of image enhancement or denoising, GAN-based solutions with adversarial training are better at constructing manifolds closer to the target data than MSE-based solutions and focus on perceptual quality rather than optimizing only for quantitative metrics [11]. This has been shown to produce more cognitively compelling results [3]. Thus, several studies have shown that GAN models are able to achieve high performance in CT denoising [3,4,12]. 

GAN consists of two models: a generative model and a discriminative model. Researchers employ various CNN structures and explore different loss functions to enhance the performance of both the generator and discriminator models in denoising tasks. WGAN-VGG-GP [3], which changes the objective to Wasserstein-1 distance to solve the problem of poor learning of Vanila GAN and introduces gradient penalty to the WGAN-GP model [12] with VGG to measure perceptual differences, has shown great results in GAN-based CT Denoising research. It has been reported to achieve PSNR values of 23.39 and 22.16 with SSIM scores around 0.79, while also providing superior visual quality compared to both MSE-based and traditional GAN models [3]. SAGAN [4] employed a U-Net-based generator model and PatchGAN [13]-based discriminator. Further, it tried to solve the problem of smoothing the edges of structures in the output of the GAN-based denoising model through sharpness loss. DU-GAN [12] enables adversarial training in both the image domain and the gradient domain, which reduces noise while mitigating streak artifacts. DU-GAN achieved a PSNR of 22.31, RMSE of 0.0802, and the highest SSIM score of 0.7489 on the Mayo-10% dataset, outperforming both MSE-based and other GAN-based methods. This indicates that DU-GAN not only effectively reduces noise but also preserves important structural information, as reflected in its superior SSIM scores.

Many studies employing GAN models as above have significantly improved CT denoising performance. However, obtaining paired datasets in real-world medical settings remains challenging, necessitating denoising techniques applicable to unpaired datasets.

### 2.2. Techniques for Denoising with Unpaired Datasets

To solve the problem of denoising in unpaired datasets, researchers have attempted to utilize image domain translation. CycleGAN is a representative model of image domain translation [14], which uses cycle consistency loss to learn translation between two different domains; i.e., it captures the unique features of one set of images and transfers them to another set.

Kang et al. [15] introduced CycleGAN for unpaired multiphase computed tomography angiography (CTA) denoising. GAN generates high-quality samples quickly, but it has poor mode coverage and is prone to mode collapse. However, by introducing cycle consistency loss, the researchers demonstrated that CycleGAN works well without mode collapse on unpaired CT datasets. GAN-CIRCLE [16] introduced Wasserstein distance into the framework based on CycleGAN and showed that by using unpaired datasets, the model can effectively learn more complex structured features. Cycle-free CycleGAN [17] introduced AdaIN [18], which is used in StyleGAN, and achieved efficient performance in unpaired CT denoising while reducing model complexity by using only a single invertible generator and discriminator. Yin et al. [19]. introduced an attention-gate to CycleGAN to extract useful salient features and attempted to improve denoising performance.

There has also been recent work on LDCT denoising using contrastive learning, with NDCT as positive samples and LDCT as negative samples. In CCN-CL [20], a contrastive regularization loss term was proposed and performed using a content–noise complementary learning strategy. ESAU-Net [21], which utilizes a channel-wise self-attention mechanism in its ESAU-Net, improved global and local context preservation, leading to a 15% increase in image clarity. Yuanke Zhang et al. [22] propose a bidirectional contrastive unsupervised denoising (BCUD) technique to solve the problem of difficulty in obtaining paired data between LDCT and NDCT; they use a bidirectional generator to generate synthetic images which are then used for training.

### 2.3. Recent Developments in Unsupervised Image-to-Image Translation

Translating a noisy image into a clean, high-quality image, image-to-image (I2I) translation provides new strategies for improving image quality, maintaining fine details, and handling the complexities of medical imaging data. The field of I2I translation has seen significant advances, which offer potential solutions to some of the challenges encountered in traditional convolutional neural networks (CNNs).

Recently, diffusion models have emerged as a powerful alternative to GANs for various image synthesis tasks, including medical imaging [23]. Unlike GANs, which rely on adversarial training to generate images, diffusion models work by iteratively refining a noisy image towards the target image distribution. This approach has been shown to be highly effective in generating high-quality images with fewer artifacts [24]. Arslan et al. [25] introduced a self-consistent recursive diffusion bridge for medical image translation, demonstrating superior performance in tasks that require cross-modality synthesis, such as CT to MRI conversion. Incorporating diffusion models into CT denoising frameworks could potentially enhance the quality of the generated images by further reducing noise and artifacts.

Transformers [26], known for their ability to capture long-range dependencies in data, have also been successfully applied to medical image synthesis. I2I-Mamba [27] integrates selective state–space modeling (SSM) with a transformer architecture, enabling efficient context capture without the overhead typically associated with transformers. This model outperforms traditional CNNs in tasks like MRI-CT synthesis by maintaining both local precision and global contextual understanding, which is crucial for high-fidelity image reconstruction.

## 3. Methodology

### 3.1. Framework

**Figure 1:**
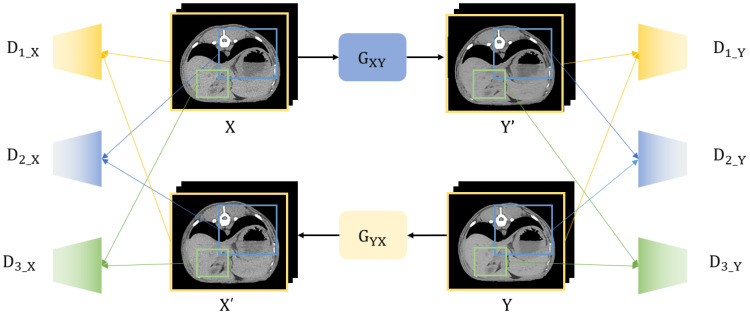
Low-dose CT denoising framework: X is LDCT; Y is SDCT. The network consists of two main components: the generator networks and the discriminator networks. The bounding boxes within the CT images are color-coded to represent different input sizes for the discriminators. The yellow boxes correspond to the $D_{1}$ discriminators, which process the image at a size of 512 × 512 pixels. The blue boxes correspond to $D_{2}$ at 256 × 256, and the green boxes represent $D_{3}$ at 128 × 128.

The overall framework of the proposed network architecture is illustrated in Figure 1. We proposed a low-dose CT denoising framework based on the CycleGAN model. We used unmatched pairs of LDCT and SDCT images. The network approaches employed in previous CycleGAN papers have certain drawbacks. One such disadvantage is the propensity to produce checkerboard artifacts attributed to deformations in the latent space. Additionally, the approach fails to adequately leverage the spatial information present in the input image. Consequently, we introduce an anti-aliasing generator and multi-scale discriminator as a remedy to address these aforementioned issues. 

#### 3.1.1. Anti-Aliasing Encoder and Decoder

The encoder incorporates spectral normalization [28], instance normalization [29], LeakyReLU [30], and a BlurPool layer. Spectral normalization controls the Lipschitz constant, serving as a regularization term to prevent sensitivity to singular directions and promote adaptive regulation of the principal gradient direction, considering features in diverse directions. Instance normalization (IN) is used to calculate the mean and variance for each input sample to keep the style of each image independent. The two normalization operations bring the benefits of stability and style preservation. After two normalizations, the image is converted to a non-linear value by passing it through the LeakyReLU and then down-sampling it through the BlurPool layer. BlurPool is one of the techniques proposed to solve the problem of aliasing during pooling operations in deep learning. According to Richard Zhang et al. [8], MaxPool [31] is divided into two steps. The max kernel extracts the maximum value within each window, and sub-sampling uses the extracted value to reduce the resolution of the output. One thing to note is that aliasing may occur in the subsampling step, which breaks the moving homogeneity. BlurPool works by blurring high-frequency components by applying an anti-aliasing filter before the sub-sampling step. 

**Figure 2:**
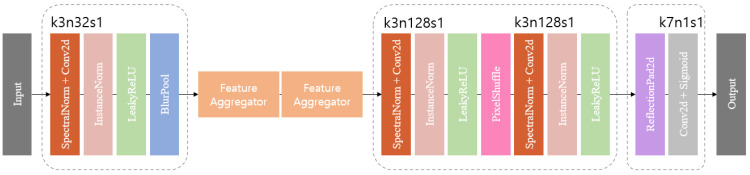
BlurPool generator structure, specifying the kernel size (k), number of feature maps (n), and stride (s) for each convolutional layer.

The decoder consists of the same sequence as the encoder: spectral norm, instance norm, and LeakyReLU, with the last layer of up-sampling using PixelShuffle [32]. PixelShuffle reduces the number of channels by 1/32 and adjusts the upscaling factor to 3. PixelShuffle works by taking pixels from each channel and arranging them in 3 × 3 groups, effectively scaling the height and width of the features by the square of scale to create a feature map in the channel axis and increasing scale by bringing the features created in the channel axis into one dimension. This process provides PixelShuffle with a significant advantage in terms of expressive power, as it possesses more network parameters compared to conventional deconvolution layers. The final stage (Figure 2) consists of a reflection padding, a convolution layer, and a sigmoid, and uses the reflection padding earlier to use a kernel size of seven in the convolution layer.

#### 3.1.2. Hierarchical Feature Aggregator

The feature extraction step in traditional CycleGAN is accomplished through U-Net [33], which consists of an encoder to compress the image into a low-dimensional representation and a decoder to expand it back into a high-dimensional representation, utilizing skip-connections to integrate both low- and high-dimensional information. However, models like PixelShuffle face challenges related to learning time and gradient vanishing problems, limiting layer depth and struggling to reproduce high-frequency components. In contrast, ResNet [31] effectively learns residuals and reproduces high-frequency components, making it frequently used in denoising tasks. According to Jie Liu et al. [34], the conventional structure of ResNet does not fully exploit the capabilities of the residual block, leading to the proposal of a residual feature aggregation (RFA) structure to better utilize local residual learning [34].

RFA was initially proposed for single-image super-resolution tasks. RFA structures aggregate residual features locally to improve the representation of fine details [35]. This method enhances the learning of high-frequency components, resulting in sharper and more detailed image reconstructions. The RFA network combines multiple residual blocks and uses hierarchical feature synthesis to retain and propagate essential details throughout the network layers [10].

In this study, the feature aggregator is based on the HFS network. The three residual blocks of the HFS are concatenated together with the output of the last residual block. Finally, a 1 × 1 convolution is applied to fuse these features with the identity features before adding them together. Unlike simply stacking multiple residual blocks, HFS leverages residual features non-locally. The hierarchical information contained in the residual blocks propagates to the end of the HFS module without loss or interference, leading to a more discriminative feature representation.

**Figure 3:**
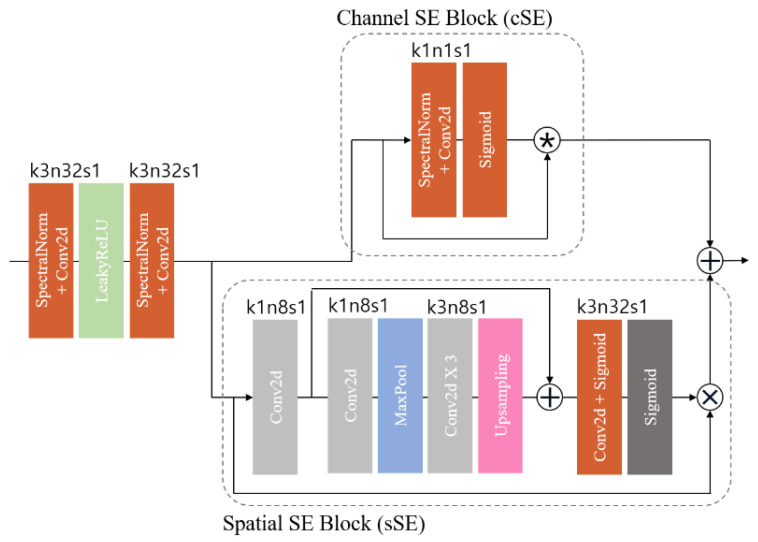
Feature attention block, specifying the kernel size (k), number of feature maps (n), and stride (s) for each convolutional layer.

To enhance the effectiveness of the HFS framework, we employed spatial and channel squeeze and excitation (SE) blocks (scSE) (Figure 3). The scSE blocks are an advancement of the squeeze and excitation (SE) block [36], which learns channel-specific descriptors accounting for spatial dependence through global average pooling. This process resizes the input feature map to emphasize useful channels. The scSE combines the channel SE (cSE) block, which squeezes spatial regions and excites channels, with the spatial SE (sSE) block, which provides spatial attention without altering the receptive field.

In medical imaging, pixel-level spatial information is crucial for accurately segmenting anatomical structures [10]. The spatial SE (sSE) block squeezes along channels and excites spatially, offering spatial attention while maintaining the receptive field. This enables the model to focus on specific regions of interest, enhancing segmentation performance. The scSE blocks, combining cSE and sSE blocks, recalibrate the feature map according to channel and space. These blocks are integrated into the residual block of the HFS module in this study.

### 3.2. Multi-Scale Discriminator Network Architecture

The discriminators in the model used the multi-scale discriminator method, which is a CycleGAN structure that performs image-to-image conversion and allows the discriminators to see the details of the image and understand and evaluate different information. 

**Figure 4:**
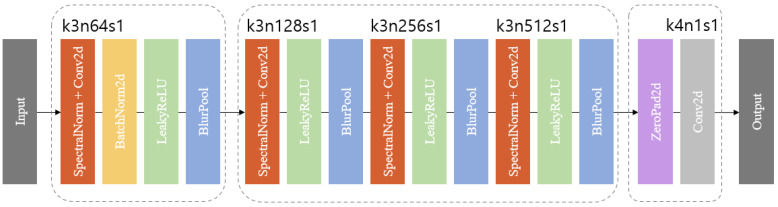
Multi-scale discriminator structure, specifying the kernel size (k), number of feature maps (n), and stride (s) for each convolutional layer.

The multi-scale discriminator is a method that converts an input image to multiple resolutions, evaluates each version independently, and then aggregates them. By evaluating in multiple resolutions, it is possible to make more accurate evaluations of edges and patterns by utilizing information not only from high-resolution images but also from low-resolution images. Each image is extracted by random sampling to create a consistent image. In Figure 4, the $D_{1}$ is 512 × 512 and $D_{2}$ is 256 × 256 and $D_{3}$ is 128 × 128 in size. 

Figure 4 shows the detail of the discriminator blocks. The first discriminator block consists of spectral normalization and convolution layers, followed by batch normalization and finally the LeakyReLU activation function and BlurPool layer. The second stage is the structure of the first discriminator block subtracted with the batch normalization. In the final stage, the ZeroPad2d layer is used to apply a convolution layer with a padding of 1 on top and bottom. The kernel size of the convolution layer is set to 4 and the stride to 1.

### 3.3. Loss Function

In this work, we use adversarial loss, cycle consistency loss, and identity loss to induce learning. Each loss is defined by Equations (1)–(3).

#### 3.3.1. Adversarial Loss

Adversarial loss aims to minimize the cross-entropy between the distributions of generated and real data while maximizing the discriminator’s ability to differentiate between them. The loss encourages the generators to produce outputs closely resembling the target domain while ensuring that the discriminator can adeptly distinguish between generated and real samples. The adversarial loss $L_{GAN}$ defined as follows:

(1) $$L_{GAN}\left(G,\;D_{i_{Y}},\;X,\;Y\right)=\frac{1}{N}\sum_{i=1}^{N}E_{y\sim P_{data}\left(y\right)}\left[logD_{i\_Y}\left(y\right)\right]+E_{x\sim P_{data}\left(x\right)}[\log\left(1-D_{i\_Y}\left(G\left(x\right)\right)\right]$$

where X and Y denote input data and target, respectively. $G\left(\cdot \right)$ takes x as input and generates fake y. $D_{i\_Y}$ is the i-th discriminator that judges the authenticity of the Y data. We utilize an additional loss that is identical, employing $D_{i\_X}$ and $F\left(\cdot \right)$. $D_{i\_X}$ is the inverse of $D_{i\_Y}$, and $F\left(\cdot \right)$ is also the inverse of $G\left(\cdot \right)$. For x, y are LDCT images and SDCT images, respectively. We set N=3.

With adversarial loss alone, the network cannot successfully learn the mapping relationship between the source and target domains, resulting in mode collapse. To deal with this issue, we applied cycle consistency loss. 

#### 3.3.2. Cycle Consistency Loss

Cycle consistency loss enables $F\left(G\left(x\right)\right)\approx x,\;G\left(F\left(y\right)\right)\approx x$, referred to as forward and backward cycle consistency, respectively. In fact, it imposes a shared latent spatial constraint to preserve the original content during the cycle reconstruction. The cycle consistency loss $L_{cyc}$ is defined as follows:

(2) $$L_{cyc}\left(G,\;F\right)=E_{y\sim p_{data}\left(y\right)}\left[\|G\left(F\left(y\right)\right)-{y\|}_{1}\right]+E_{x\sim p_{data}\left(x\right)}\left[\|F\left(G\left(x\right)\right)-{x\|}_{1}\right]$$

#### 3.3.3. Identity Loss

Adding loss of identity has several benefits. First, it preserves the content of the input images during translation, resulting in more visually consistent results. Second, it allows the model to better handle the task of translating between unmatched images by encouraging the model to maintain the structural integrity of the input images. The identity loss $L_{idt}$ is defined as follows:

(3) $$L_{idt}\left(G,\;F\right)=E_{y\sim p_{data}\left(y\right)}\left[\|G\left(y\right)-{y\|}_{1}\right]+E_{x\sim p_{data}\left(x\right)}\left[\|F\left(x\right)-{x\|}_{1}\right]$$

#### 3.3.4. Overall Joint Loss

Combining Equations (1)–(3), we obtain the overall joint loss function of the low-dose CT denoising framework, expressed as follows:

(4) $$L\left(G,\;F,\;D_{i\_X},\;D_{i\_Y}\right)=\lambda_{adv}(L_{GAN}\left(G,\;D_{i\_Y},\;I_{X},\;I_{Y}\right)+L_{GAN}\left(F,\;D_{i\_X},I_{X},\;I_{Y}\right))+\lambda_{\mathrm{cyc}}L_{cyc}\left(G,\;F\right)+\lambda_{\mathrm{idt}}L_{idt}\left(G,\;F\right)$$

where λ is a weighting parameter to control the trade-off between adversarial loss, cycle consistency loss, and identity loss. $\lambda_{adv}$ = 0.1, $\lambda_{cyc}$ = 10.0, $\lambda_{idt}$ and = 2.0. The primary reason for this setup was to align the scales of each loss function. The $L_{GAN}$ tends to have a larger value compared to the $L_{cyc}$ and $L_{idt}$, so we set $\lambda_{adv}$ to a lower value to appropriately adjust the scale. Moreover, to minimize artifacts and distortions that could occur during the image reconstruction process, we selected hyperparameters that relatively increased the $L_{GAN}$ and $L_{cyc}$. This ensured that the reconstructed images maintained structural consistency while providing visually realistic results.

## 4. Experiments

### 4.1. Datasets

**Figure 5:**
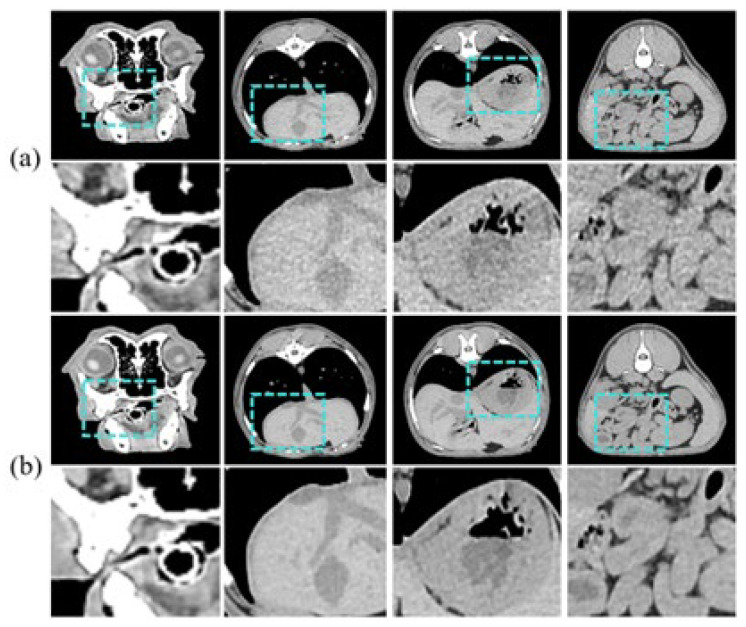
Image samples for training, showing diverse regions/tissues: (**a**) is LDCT and (**b**) is SDCT.

In this study, we used unmatched pairs of LDCT and SDCT images of 10 clinically healthy dogs provided by the Animal Hospital of Konkuk University. We observed ethical considerations, and Institutional Review Board (IRB) approval was obtained for the animal experimentation. The training data consists of 3458 axial slices of LDCT and SDCT images of 9 dogs, respectively, and the test dataset consists of 941 axial slices of one dog. Figure 5 is an example of an input sample. The CT images are grayscale and have a pixel size of 512 × 512. Coronal currents were acquired using 30 mA and 150 mA for LDCT and SDCT, respectively.

### 4.2. Evaluation Framework

The evaluation involves two key aspects. First, precision measures the proportion of the generated image that aligns with the true image sample distribution. It essentially quantifies how accurately the generated image depicts the true image sample. Recall is the proportion of real-world footage that belongs to the distribution of generated footage samples, and recall is how much of the real-world footage is reproduced by the generated footage. 

The current calculation method relies on relative density, which poses limitations in properly addressing phenomena such as densely packed generated images due to mode decay or truncation. Therefore, in this study, we introduce an improved precision and recall calculation method to address these shortcomings. The improved precision and recall [37] uses the middle layer of the InceptionV3 model [38] to extract the features of the generated images and the real images.

The regions of the real and generated images are then approximated by hyperspheres using the K-nearest neighbor method. Equation (5) gives the improved precision, which is the binary value of whether or not the generated data is present in the Kth closest region of the real image to the generated data and averaged. Equation (6) gives the improved recall, which is a binary value that averages over whether or not the actual data is present in the Kth closest region of the training data. Equation (7) is defined to compare the distance between feature vectors and is a functional expression that outputs 1 if a vector closer than the Kth feature vector exists and 0 otherwise.

(5) $$Improved\;Precision\left(\Phi_{r},\Phi_{g}\;\right)=\frac{1}{\Phi_{g}}\sum_{\phi_{g}\in \Phi_{g}}f\left(\phi_{g},{\;\Phi }_{r}\right)$$

In the following way, recall is quantified by querying for each real image whether the image is estimated manifold generated images.

(6) $$Recall\left(\phi ,\;\Phi \right)=\left\{\begin{array}{c} 1,\;if\;\|\phi -{\phi^{\prime }\|}_{2}\;\leq \;\|\phi^{\prime }-NN_{k}\left(\phi^{\prime },\;\Phi \right){\|}_{2}\;\\ for\;at\;least\;one\;\phi^{\prime }\in \;\Phi \\ 0,\;otherwise \end{array}\;\right.$$

where $\Phi_{r}{\;\mathrm{and}\;\Phi }_{g}$ are the set of features of the real image and the generated image, respectively, and $\phi_{r}\;\mathrm{and}\;\phi_{g}$ are the feature elements of the set $\Phi_{r}\;\mathrm{and}\;\Phi_{g}$ are the feature elements of $NN_{k}$. The set Φ and outputs the feature vector that is the Kth closest to the feature vector $\Phi^{\prime }$ and outputs the Kth closest feature vector to the feature vector given as input to the function.

Additionally, there are two main approaches to validating model outcomes: k-fold cross-validation and single validation. While k-fold cross-validation is generally a useful technique for assessing model performance, it has certain drawbacks. In the case of sensitive medical imaging data, there is a risk of overestimating performance if slices from the same patient are mixed between the training and validation sets during the data splitting process. To address this, a patient-based k-fold cross-validation can be considered. However, if certain pathological features are unique to specific patients, this approach may introduce data imbalance across folds, leading to distorted performance evaluation. Moreover, dividing the data into multiple folds and training and evaluating the model on each fold is computationally intensive, significantly increasing training time. Therefore, in this study, we conducted an independent single validation. We aimed to enhance the reliability of the evaluation by using improved precision and recall metrics over traditional methods that rely on relative density. An appropriate validation split ratio of approximately 21% was used.

### 4.3. Implementation Details

In Equation (7), which is used to evaluate precision and recall for the generated images, K is 3. The data processing for precision and recall evaluation is as follows. When extracting image patches, the outside of the CT cylinder is fixed to a value such as −2048, so we set an ROI inside the cylinder and randomly sampled it with a size of 128 × 128. In the process, if the sampled patches contained many parts that were not the organs we wanted to see, we excluded them and resampled them. 

We used min-max normalization to standardize the characteristics of the images to make consistent comparisons. In Equation (8), X is the value of each pixel in the original image and X′ is the normalized value. This ensures that the pixel values in the image are represented within a certain range.

(7) $$X^{\prime }=\frac{X-\min\left(X\right)}{\max\left(X\right)-\min\left(X\right)}\;$$

When training the network, we used Adam [39] as the optimizer and set $\beta_{1}$ = 0.9, $\beta_{2}$ = 0.999, and the learning rate was fixed at $1\times 10^{-4}$. The key parameters used in training the CNN are provided in each figure. Weight initialization was performed using the Xavier uniform method. The training was performed on a single RTX GeForce 4090 with a batch size of 1 and input size of 512 for 5000 epochs.

In the selection of hyperparameters, consistent performance improvements were observed at specific values through experimentation. Although the chosen hyperparameters were not fully optimized due to computational constraints, they were found to be practical and effective configurations, as evidenced by the empirical performance observed in the experiments.

### 4.4. Ablation Study on Model Architecture

**Figure 6:**
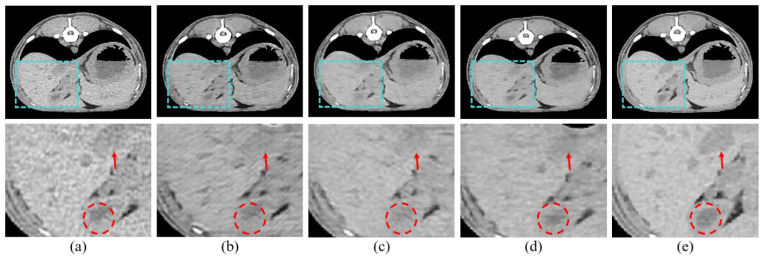
Ablation studies of the proposed method, (**a**) LDCT, (**b**) cSE alone, (**c**) sSE alone, (**d**) scSE, and (**e**) SDCT.

We adopted scSE blocks to maximize the performance of the HFS modules. In this section, we performed three ablation studies to validate the effectiveness of scSE. We compared cSE alone, sSE alone, and the scSE method, which combines cSE and sSE. Each method was evaluated based on whether it applied spatial squeeze and channel excitation or channel squeeze and spatial excitation, and we identified and described the features of each approach. First, cSE alone emphasized useful channels by squeezing them along spatial regions and excitations along channels. The results are shown in Figure 6b and indicate reduced noise and improved perceptual quality compared to the conventional LDCT image. However, the contrast is still not sharp enough, and important structures are faintly visible. Second, sSE alone emphasizes pixel-wise information by spatially exciting them along the channel, as opposed to cSE. The results, shown in Figure 6c, demonstrate improved contrast, making anatomical structures more distinct. However, the reconstructed high-resolution image appears uneven and less smooth. Finally, the scSE method combines the advantages of both cSE and sSE. Figure 6d shows that scSE yields the most pronounced contrast, improving upon the limitations observed with sSE alone.

The quantitative evaluation of each item is shown in Table 1. Precision and recall are evaluated by truncation 1.0 and 0.3. Truncation is a technique used to sample the best images from the trained distribution after training the GAN with the truncation trick. The farther away from the mean of the distribution, the more transformed images will be sampled; close to 0 means sampling near the mean, and close to 1 means sampling from the distribution as it is. Low and standard dose are scores for comparison between the different methods. The scores when applying cSE, sSE, and scSE to the base model show positive results, indicating that LDCT can be used as input data to produce images close to SDCT. The highest and next highest scores in each area are shown in bold and underscore, respectively. scSE yielded the highest values for precision and recall. Precision was next highest with cSE, and recall was next highest after that with sSE. The proposed scSE method was found to be the most effective in both quantitative and perceptual evaluation.

### 4.5. Comparison of Different Methods

For the purpose of comparing noise reduction performance, we employed three distinct deep learning models. RED-CNN [40], rooted in residual learning, enhances resolution by extracting features from high-resolution images at each layer and adding these features to the output of the previous layer. We utilized HFS to compare its effectiveness in feature extraction from images. WGAN-VGG [3] is a model based on the Wasserstein GAN (WGAN) [41], which improves the stability of GANs and the quality of generated images. We used it to compare with the anti-aliasing and stability effects of BlurPool and the regularization term that we used. Lastly, DU-GAN utilizes a gradient domain discriminator to enhance edge information and reduce streak artifacts caused by photon starvation. It focuses on increasing edge sharpness and reducing streak artifacts to generate high-resolution images. Therefore, we chose this model for comparison with the multi-scale discriminator.

**Figure 7:**
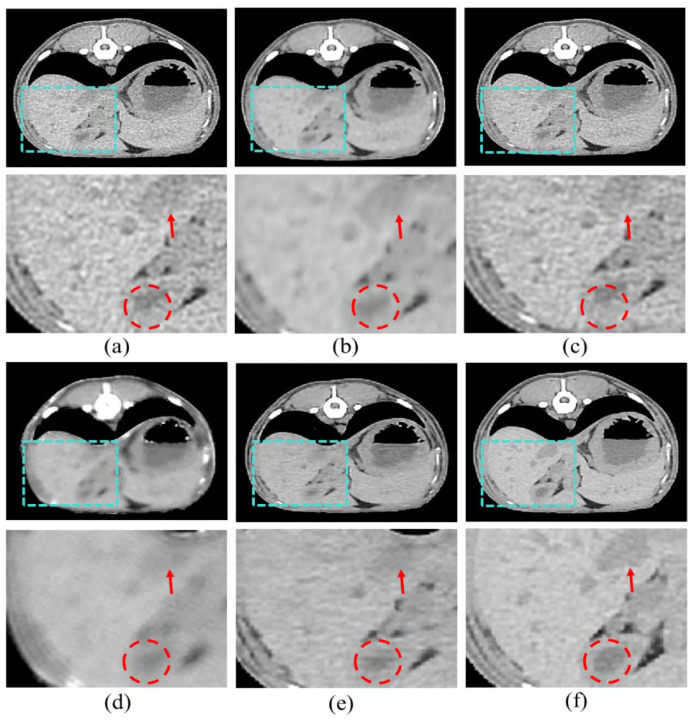
Results of the proposed model and other models: (**a**) LDCT, (**b**) RED-CNN, (**c**) WGAN-VGG, (**d**) DU-GAN, (**e**) Ours, and (**f**) SDCT.

Figure 7 shows the resulting images of the proposed model and the comparison models. Figure 7b is the result image for RED-CNN, indicating significant noise reduction compared to the LDCT images, though sharpness is still lacking. Figure 7c shows the result for WGAN-VGG, which is sharper, but shows less noise reduction. Figure 7d shows the results for DU-GAN, where the edges are cleaner and the denoising is effective, but sharpness is still an issue. Figure 7e shows our proposed model, which addresses the shortcomings of the previous models and best represents the features of SDCT.

## 5. Discussion

In this study, we introduced a CycleGAN-based denoising method tailored for low-dose CT (LDCT) imaging, specifically targeting animal LDCT. This innovative approach surpasses conventional CT denoising methods, offering enhanced image quality while concurrently minimizing radiation exposure, thereby showcasing its potential in veterinary imaging. The key is the cohesive integration of several techniques adept at handling noise and deformation, critical aspects in medical image reconstruction. The utilization of BlurPool and regularization terms effectively mitigates distortion in high-resolution images, while PixelShuffle enhances expressiveness in the output. Additionally, the HFS structure and scSE block retain and emphasize features, facilitating the accurate reproduction of contrast and vital organs in the resulting image. The generator’s efficacy in producing realistic images heavily relies on the effectiveness of the discriminator, for which we introduce a multi-scale discriminator. This novel approach ensures accurate assessments of edges and patterns by incorporating information from various resolutions, thus effectively balancing the adversarial learning process and leading to improved overall performance. 

Our results demonstrate that our proposed model outperforms comparative methods in both perceptual and quantitative evaluations. In our study, the results show that our model outperforms the comparative methods in both perceptual and quantitative evaluations in Figure 7 and Table 2. In addition, in Figure 6 and Table 1, the effectiveness of the proposed scSE method for denoising LDCT images in animal imaging was demonstrated. Through three ablation studies, we compared the performance of cSE alone, sSE alone, and the combined scSE method, revealing distinct advantages and limitations associated with each approach. The successful implementation of the scSE method underscores the importance of innovative approaches in addressing the challenges associated with LDCT imaging in veterinary medicine.

Our method offers a promising solution for improving diagnostic accuracy and patient care in veterinary imaging. 

While our proposed method demonstrates greater performance in improving image quality compared to other methods, there is still room for improvement in terms of contrast and sharpness when compared to SDCT. In the future, there is potential for further enhancement of our denoising method and exploration of its applicability across diverse animal species and imaging modalities. This could involve refining existing components to improve the efficacy and efficiency of LDCT denoising in veterinary imaging, exploring the generalizability of our approach, and conducting real-world clinical validation to provide valuable insights into its practical utility and transformative impact on animal healthcare. Additionally, our target was the pre-contrast LDCT images. In this case, applying our model to LDCT images effectively reduced radiation exposure by producing images with noise levels comparable to SDCT images. However, when our model was applied to post-contrast LDCT images, it did not achieve the same level of quality as SDCT images. This is likely because post-contrast images exhibit greater contrast between areas with and without contrast agent, resulting in more distinct image features. This increased contrast can introduce complex and challenges in noise reduction, making it more difficult to achieve consistent results.

Deep learning tasks on medical images inherently face reliability challenges. While we have shown the reliability and perceptual superiority of the proposed low-dose CT denoising framework through precision and recall evaluation methods, there are still doubts about its practical clinical applicability, as it is not based on pixel-wise evaluations such as PSNR or SSIM. However, due to the unpaired nature of the dataset, employing this method remains challenging. As deep learning advances into real-world applications, it is clear that different evaluation methodologies must be considered. 

## 6. Conclusions

In this paper, we propose a method to apply deep learning to LDCT to make it as good as SDCT. We call this method the low-dose CT denoising framework. The proposed framework is based on CycleGAN and proposes an anti-aliasing generator and multi-scale discriminator with various proven techniques. By overcoming the limitations of the existing CycleGAN for input images, the proposed framework outperforms existing models in both quantitative and perceptual evaluations in terms of preserving anatomical information and suppressing image noise. With a framework that can generate high-quality images under low-dose conditions, we expect to reduce radiation exposure to animals and make future animal CT imaging safer.

The CT dataset used in this study is sufficient to produce results, but we plan to accumulate more data and conduct various experiments on the proposed framework to extend it to various fields and tasks in the future.

## Figures and Tables

**Table 1:** Precision and recall evaluation of the proposed methods in ablation studies: The highest and next highest scores in each area are shown in bold and underline, respectively.

Ablation Term	Precision		Recall	
Truncation	1.0	0.3	1.0	0.3
Low and standard dose	0.45	0.45	0.66	0.37
Base model + cSE	0.88	0.91	0.90	0.88
Base model + sSE	0.91	0.91	0.9	0.9
Base model + scSE	**0.93**	**0.94**	**0.92**	**0.91**

**Table 2:** Comparison model precision and recall evaluation: The highest and next highest scores in each area are shown in bold and underline, respectively.

Ablation Term	Precision		Recall	
Truncation	1.0	0.3	1.0	0.3
Low and standard dose	0.45	0.45	0.66	0.37
RED-CNN [40]	0.86	0.83	0.91	**0.93**
WGAN-VGG [3]	0.71	0.70	0.82	0.85
DU-GAN [12]	**0.94**	0.93	0.87	0.84
Proposed	0.93	**0.94**	**0.92**	0.91

## Data Availability

The data presented in this study are available on request from the corresponding author.
